# From CAPTCHA to Commonsense: How Brain Can Teach Us About Artificial Intelligence

**DOI:** 10.3389/fncom.2020.554097

**Published:** 2020-10-22

**Authors:** Dileep George, Miguel Lázaro-Gredilla, J. Swaroop Guntupalli

**Affiliations:** Vicarious AI, San Francisco, CA, United States

**Keywords:** Recursive Cortical Network, AGI, generative model, neuroscience inspired AI, biologically guided inductive biases

## Abstract

Despite the recent progress in AI powered by deep learning in solving narrow tasks, we are not close to human intelligence in its flexibility, versatility, and efficiency. Efficient learning and effective generalization come from inductive biases, and building Artificial General Intelligence (AGI) is an exercise in finding the right set of inductive biases that make fast learning possible while being general enough to be widely applicable in tasks that humans excel at. To make progress in AGI, we argue that we can look at the human brain for such inductive biases and principles of generalization. To that effect, we propose a strategy to gain insights from the brain by simultaneously looking at the world it acts upon and the computational framework to support efficient learning and generalization. We present a neuroscience-inspired generative model of vision as a case study for such approach and discuss some open problems about the path to AGI.

## 1. Introduction

Despite revolutionary progress in artificial intelligence in the last decade, human intelligence remains unsurpassed in its versatility, efficiency, and flexibility. Current artificial intelligence, powered by deep learning (LeCun et al., 2015; Schmidhuber, 2015), is incredibly narrow. For each task that needs to be tackled, one has to laboriously assemble and label data, or spend an enormous amount of computational power to let the system learn through trial and error. Compared to humans, even extremely simple tasks require orders of magnitude more data to train, and the performance of the trained systems remains way too brittle (Lake et al., 2016; Kansky et al., 2017; Marcus, 2018; Smith, 2019). For these reasons, today's AI systems are considered to be narrow, while human intelligence is considered to be general. What would it take to build an artificial general intelligence (AGI)?

To build AGI, we need to learn the principles underlying the data efficiency of the human brain. The need for this can be argued from the viewpoint of the *No Free Lunch* theorem (Wolpert and Macready, 1997). An algorithm's efficiency at learning in a particular domain comes primarily from the assumptions and inductive biases that the algorithm makes about that domain, and no single algorithm can be efficient at all problems. The more assumptions an algorithm makes, the easier learning becomes. However, the more the assumptions, the fewer the number of problems that can be solved. This means that generality and efficiency of the brain has to be limited to certain class of problems—the kinds of problems humans are good at solving efficiently with their current sensory apparatus, and whatever generalizations could be derived from those principles to other domains with the use of novel sensors (Bengio and LeCun., 2007; George, 2008; Locatello et al., 2019).

To build machines with general intelligence, the question we need to ask is this: What are the basic set of assumptions that are specific enough to make learning feasible in a reasonable amount of time while being general enough to be applicable to a large class of problems? Our brain is proof that such a set of assumptions exists. Looking into the brain helps to speed up our search for the *Goldilocks* set of inductive biases, and to tease apart the interdependent representational scaffolding by which they need to be operationalized in a machine (George, 2017).

But just how should one look into the brain in search of inductive biases and principles of general intelligence? Which brain should one look into first? Should we start from simpler creatures, like worms and flies, and work our way up to humans? Even for mammalian brains, there is a bewildering array of experimental findings in neuroscience, scaling several levels of investigation from single neuron physiology to microcircuits of several hundred cells to psychophysical correlates of intelligence spanning several brain areas. It is not clear which of these insights are relevant for machine learning and artificial intelligence because some of the observations might relate to the implementation substrate, or arbitrary constraints on the amount of hardware. To be of use, we need principles that are relevant for information processing (Marr, 1982). In this paper we will address this problem and describe a systematic process by which we can look at the brain for insights into building general intelligence.

First, we will describe an evolutionary perspective through which we could view the contemporary advancements and the path to general intelligence. Then, we describe why common sense is the holy grail of general intelligence and how even perception and motor systems need to be considered in concert with the goal of achieving common sense. Looking into the brain should be from the point of gaining insights regarding the innate biases and representational structures needed to efficiently acquire, and manipulate commonsense knowledge while interacting with the environment. We then describe a systematic “triangulation” method for looking at the brain for these kinds of insights. We exemplify this method using our work on Recursive Cortical Network (RCN) (George et al., 2017), a generative vision model that is built according to these principles. We then discuss a few open questions regarding general intelligence before offering closing thoughts.

## 2. General Intelligence: An Evolutionary Perspective

### 2.1. Direct Fit on Isolated Tasks Does Not Produce General Intelligence

From the origin of life circa 650 million years ago to now, evolution has produced creatures of varying levels of complexity and adaptive behavior. These behaviors are controlled by sensors and circuits that were tuned for fitness over many generations (Schneider, 2014). These building blocks are subsequently reused to form of new, more complex organisms with more intricate mechanisms. The result of this is a host of organisms that are precisely tuned to the niches they live in. A frog is exceptional at catching flies, and geckos are very adept at climbing walls. However, most of these creatures rely on simple stimulus-response mappings for their behaviors, without a need for intricate internal models of the world they live in.

While these organisms exhibit sophisticated behavior powered by intricate circuits, each circuit, co-evolved with a particular sensory apparatus, is idiosyncratic. Reverse engineering those circuits might only reveal the clever and efficient implementations of specific functions rather than general principles of intelligence. This situation is similar to reverse engineering a highly specialized application specific integrated circuit, or highly optimized code^1^. However complex, these specialized circuits are not the seat of general intelligence.

Most of the credit for the flexible intelligence exhibited by humans and other mammals go to the newest evolutionary addition to our brains—the neocortex (Rakic, 2009). Necortex, in conjunction with old brain circuits like thalamus and hippocampus, allows mammals to build rich models of the world that support flexible behavior under various task demands. In the context of evolutionary history, neocortex is a recent event. Animals with neocortex were not even the most dominant ones on earth. For the longest time, dinosaurs dominated the earth and mammals were relegated to a nocturnal niche, from which they expanded only after the extinction of dinosaurs (Maor et al., 2017)^2^.

The evolutionary history of general intelligence has many parallels to the current situation in artificial intelligence. Deep learning can be used to train the parameters of large multi-layer artificial neural networks to map training data to desired labels or actions. This is analogous to how evolution created different animals. Just like evolutionary algorithms, gradient descent is a general algorithm that can be used to fit the parameters of a function approximator so that it can interpolate to represent its stimulus space well (Hasson et al., 2020). Networks trained using deep reinforcement learning and large amounts of data can learn specific stimulus-response mappings that enable them to outperform humans in specific versions of video games, but struggle when the game is even slightly altered.

Each deep learning network trained for an application can be thought of as an organism trained for its own niche, exhibiting sophisticated-looking behavior without rich internal models. The lesson from evolutionary history is that general intelligence was achieved by the advent of the new architecture—the neocortex—that enabled building rich models of the world, not by an agglomeration of specialized circuits. What separates function-specific networks from the mammalian brain is the ability to form rich internal models that can be queried in a variety of ways (Hawkins and Blakeslee, 2007; Buzsaki, 2019).

Compared to the diversity of old-brain circuits in different animals, the neocortex is largely a uniform laminated sheet of cells divided into ontogenetic and functional columns. Arguably, cortical columns can be considered as basic functional module that is repeated throughout the cortex (Mountcastle, 2003). Although regional and functional specializations exist, the expansion in motor and cognitive capabilities of mammals and humans have been largely achieved through an expansion of the neocortical sheet (Rakic, 2009). The uniformity of the neocortex gives support to the idea that a common set of principles can be found to create general intelligence (Hawkins and Blakeslee, 2007; Harris and Mrsic-Flogel, 2013). Functionally, the neocortex, in combination with the hippocampal system is responsible for the internalization of external experience (Buzsaki, 2019), by building rich causal models of the world (Pearl and Mackenzie, 2018). In humans and other mammals, these models enable perception, action, memory, planning, and imagination.

### 2.2. Common Sense Is the Holy Grail

Building rich models of the world and being able to query that in context-appropriate ways is a requirement for general intelligence. From the moment we are born, we begin using our senses to build a coherent model of the world. As we grow, we constantly refine our model and access it effortlessly as we go about our lives. If we see a ball rolling onto the street, we might reason that a child could have kicked it there. When asked to pour a glass of wine, we would not search for a bottle opener if the wine is already in the decanter. If we are told, “Sally hammered the nail into the floor,” and asked whether the nail was vertical or horizontal, we can imagine the scenario with the appropriate level of detail to answer confidently: vertical. In each of these cases, we are employing our unrivaled ability to make predictions and inferences about ordinary situations. This capacity is what we call common sense (Davis and Marcus, 2015).

Common sense arises from the distillation of past experience into a representation that can be accessed at an appropriate level of detail in any given scenario. Although commonsense is largely treated as a language understanding problem, a large portion of the required knowledge is non-verbal and stored in our visual and motor cortices to form our internal model of the world (Lee, 2015). For common sense to be effective it needs to be amenable to answer a variety of hypotheticals—a faculty that we call imagination. This leads us to causal generative models (Pearl and Mackenzie, 2018), and inference algorithms (Pearl, 1988) that can query these models flexibly (Lázaro-Gredilla et al., 2020). Insights from the brain can help us understand the nature of these generative models and how to structure them for efficient learning and inference.

## 3. The Triangulation Strategy for Learning Lessons From the Brain

Neuro and cognitive sciences produce a vast array of data every year. It is natural for a machine learning researcher to get intimidated by this complexity and conclude that nothing can be learned from the brain that is of value to artificial intelligence. Similarly neuroscientists who want to understand the brain could feel disheartened by the variance in the experiments.

**Figure 1:**
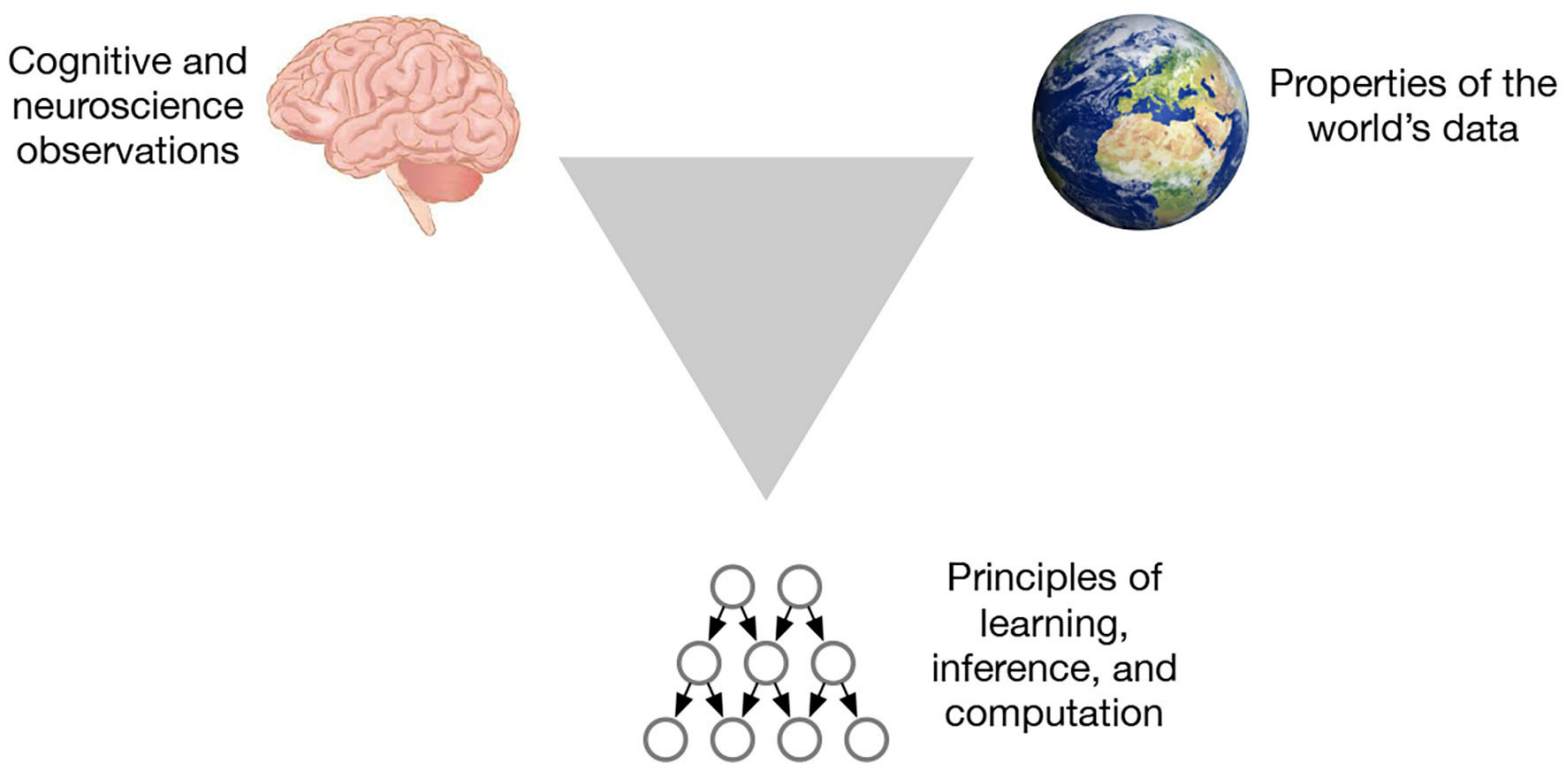
The triangulation strategy for extracting principles from the brain looks at three aspects at the same time.

We believe there is a systematic way to overcome these barriers to extract principles that are relevant for learning and inference, while also understanding cortical circuits from the view of information processing. The trick is to investigate three aspects at the same time: the brain, the world, and the computational framework (Figure 1). The world is not random. Laws of physics determine how the world is organized, and the structure of the brain circuits is tuned to exploit the regularity of this world (Simoncelli and Olshausen, 2001). The match between the brain and the world also has algorithmic advantages (Conant and Ross Ashby, 1970). The triangulation strategy is about utilizing this world-brain-computation correspondence: When we observe a property of the brain, can we match that property to a an organizational principle of the world? Can that property be represented in a computational framework to produce generalizations and learning/inference efficiency? If we find an observation that can be explained from all three angles, we can be reasonably confident that we have found a property that is useful.

To further determine whether a property is useful, it has to be incorporated into a model that solves problems from the world. Solving real-world problems that the brain can solve is another way in which we establish correspondence between all the three corners of the triangle. While solving real-world problems, it is important to test for the characteristics of the neocortex—model-building, data-efficiency, and strong generalization. If we were correct in our hypotheses about the inductive biases learned from the brain, it would be verified by testing for these properties in the real-world task performance of the model.

This triangle also helps us understand the different kinds of models researchers build. Computational neuroscience models often deal with just the observed phenomena from the brain, often missing the connections with properties of the world, or algorithmic principles, or both. Pure machine learning models deal with only two vertices of the triangle, ignoring insights that might be learned from the brain.

In the coming sections we will use this framework to analyze the set of assumptions we used in our work on Recursive Cortical Networks (RCN) (George et al., 2017), which is a neuroscience-guided generative vision model that we developed. We argue that simultaneously considering all three vertices of the triangle is fruitful and advantageous.

## 4. Recursive Cortical Network: A Vision Model Grounded in Principles Learned From Cognitive Science and Neuroscience

### 4.1. What Kind of Visual Generative Model Is Suitable for Common Sense?

Common sense requires storing a large amount of knowledge about our world in our visual and motor systems and then recalling those in the appropriate moments in the appropriate level of detail. Consider again the sentence “Sally hammered a nail into the floor,” and how you arrived at the answer for the question whether the nail was horizontal or vertical. People answer this query by simulating the scenario of Sally, the floor, and the action of pounding using a hammer, and then retrieving the answer from the simulation (Zwaan and Madden, 2005). Although the question and answer are presented in natural language, most of the information for performing this simulation are in the visual and motor systems. Answering the above query is a typical example of common sense.

To support common sense, our perceptual and motor experience need to be abstracted to concepts (Lázaro-Gredilla et al., 2019) and linked to language, as described by Barsalou in his work on perceptual symbol systems (Barsalou, 1999). According to Barsalou, in addition to the usual tasks of object and activity recognition, segmentation, reconstruction, etc., typically associated with a visual system, for a visual generative model need to have the following characteristics to support concept formation and commonsense:

- Componential and compositional: The generative model should be componential and compositional as opposed to holistic and monolithic. Photo realistic image generation is not the goal of this generative model. Instead, the generative model should allow for composing different elements of a scene—the objects, object-parts, and backgrounds in different ways.
- Factorized: The generative model should have factorized representations for different aspects of objects, backgrounds and interactions. An example of factorization is shape and appearance, or contours and surfaces.
- Hierarchical: The model contains multiple layers with identical structure, with higher layers being formed by the aggregation of pieces of the lower layers, in a recursive way.
- Controllable: The generative model should allow for top-down manipulation of its different components.
- Flexible querying, and inference to best explanation: The generative model should be able to perform inference to best explain the evidence in the scene. Moreover, the generative model should support flexible querying, not just the type of query it was trained to answer.

Typical computer vision work often focuses on optimizing for a particular query like classification, or segmentation. Even when an underlying feature set is reused, the inference networks are different for the different queries. Our goal in building RCN (George et al., 2017) was different—we wanted to build a probabilistic model on which recognition, segmentation, occlusion reasoning, curve tracing etc., are different queries on the same model and can be answered without specifically amortizing a neural network for the particular query. This meant simultaneously satisfying many functional requirements (Table 1), and multiple tasks (Figure 5) instead of optimizing for a single query-dependent objective. It is an encouraging sign that many more recent models (Linsley et al., 2018; Kietzmann et al., 2019; Yildirim et al., 2020) have started incorporating insights from neuroscience toward building a unified general model for vision.

**Table 1:** Biological features and their computational counterparts that were simultaneously considered in the development of the RCN visual generative model.

**Biological feature**	**Computational/algorithmic reason**	**Representation in RCN**
Blobs and interblobs	Curvelet-like smoothness of natural signals, an example of which is contour-surface factorization	Structure of the contour-surface factor
Lateral connections between inter-blob columns	Higher-order contour-continuity in natural signals	Cloned structure of lateral connections for higher-order interactions.
Object-based top-down attention	Compositionality, modularity	Only positive weights. Object-background factorization
Hierarchy	Efficient learning and inference	Hierarchically structured
Border-ownership coding	Required when objects are represented in a factorized and hierarchical manner	Two clones of each feature for border-ownership coding
Feedback connections	Inference requires explaining away when the representation is compositional	Message-passing algorithms automatically do explaining away
Different dynamics for contour and surface features	Convergence of message-passing depends on the schedule	Biologically inspired message-passing schedule works better

We now consider the different properties of RCN from the viewpoint of triangulation strategy where we describe their neuroscience origins, their correspondence with the world, and their computational underpinnings.

### 4.2. Shape Bias and Factorized Representation of Contours and Surfaces

#### 4.2.1. Biological Observation

**Figure 2:**
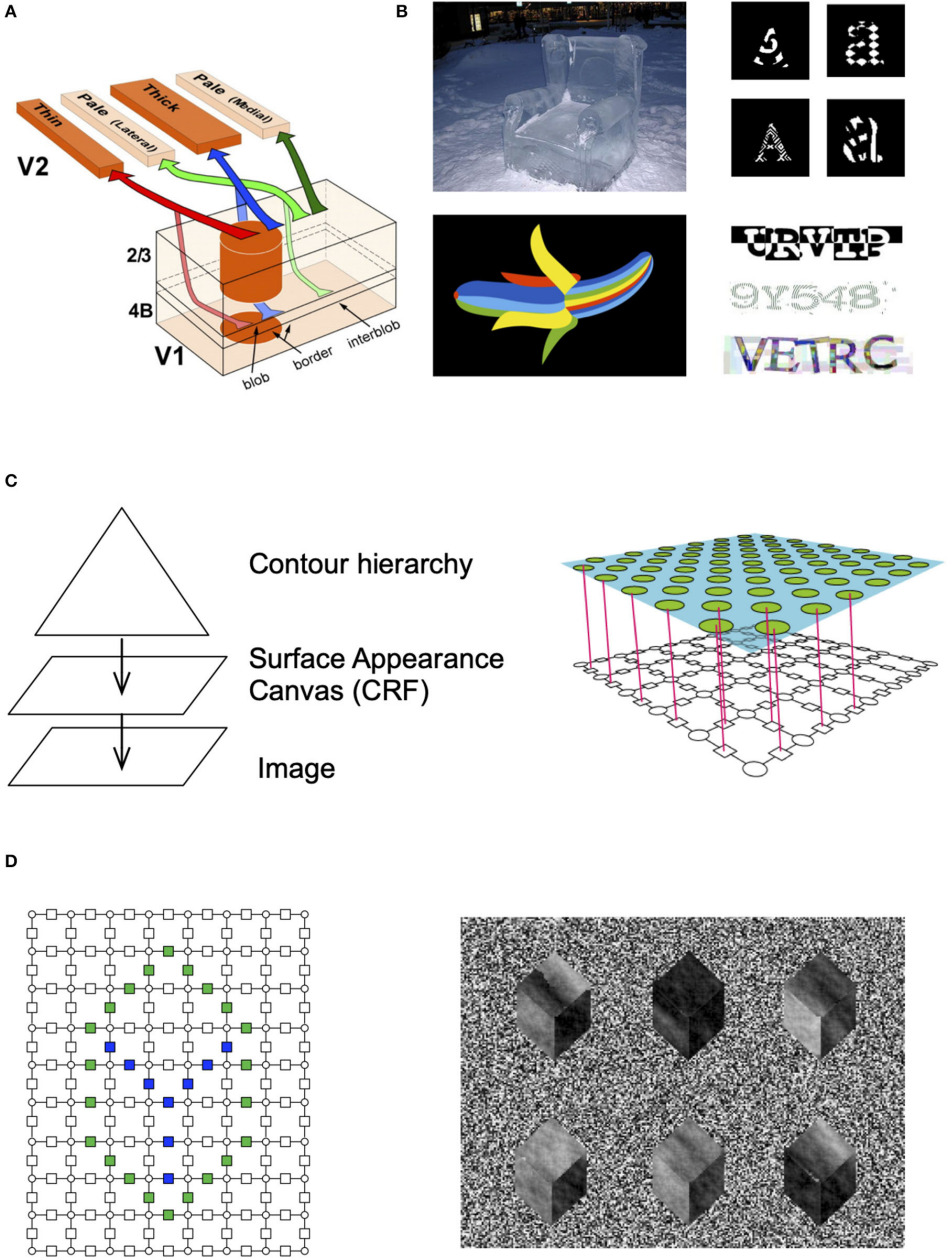
Contour-surface factorization. **(A)** The primary visual cortex has columns that are divided into blobs and interblobs, and the segregation remains in how they project to V2 (image credit: Federer et al., 2009). **(B)** People can recognize objects with unusual appearances, even when they are exposed to it for the very first time. **(C)** RCN consists of a contour hierarchy and a surface model. The surface model is a CRF. The factors between different surface patches encode surface similarity in the neighborhood and those are gated by the contour factors. **(D)** Different surface patterns can be generated by instantiating a particular set of contours, and then sampling from the surface model.

The ventral visual pathway that is responsible for object recognition and segmentation is known to be organized in parallel interacting streams (Figure 2A) called blobs and inter-blobs in the primary visual cortex, and stripes and inter-stripes in the secondary visual cortex (DeYoe and Van Essen, 1988; Felleman and Van Essen, 1991). Blobs and inter-blobs are inter-digitated cortical columns where blobs represent unoriented surface patches and inter-blobs predominantly represent oriented contours (Livingstone and Hubel, 1988; Shipp, 1995). This segregation persists into V2 and beyond (Shipp and Zeki, 1989; Zeki and Shipp, 1989). What would be the computational underpinnings and real-world correlates of this observation?

Psychophysically, it is known that humans can recognize shapes even if the surface appearances are altered significantly from their canonical appearances. For example, people can readily identify a banana that is made of rainbow colors or a chair made of ice on their first encounters of those unusual appearances (Figure 2B). Children exhibit a shape bias that make them recognize shapes irrespective of appearance. The neurobiological and psychophysical data points to a factorized representation of shape and appearance (Kim et al., 2019). Children can recognize line drawings of objects without being explicitly trained for those (Hochberg and Brooks, 1962).

#### 4.2.2. Property of the World

Is this kind of factorization a general principle that helps in many situations or is it a vision-specific hack? It turns out that cartoon + texture image decomposition is an idea suggested in image processing research for the compression and restoration of natural images (Buades et al., 2010). This idea relies on the observation that images are piece-wise smooth in two dimensions with patches of same appearance, and their discontinuities are contours. More generally, studies by Chandrasekaran et al. (2004) have suggested that natural signals, which include images, videos, speech, shockfronts, etc. share smoothness constraints that are similar, and can be encoded as a piecewise smooth function. More precisely, each piece or *surflet* is a so-called horizon function, defined as

$$f\left(x\right)=\{\begin{array}{cc} 1 & \text{if }b\left(y\right)\geq x_{N}\\ 0 & \text{if }b\left(y\right)<x_{N} \end{array}\text{ with functions }b:{\left[0,1\right]}^{N-1}$$

$$\to \left[0,1\right]\text{ and }f:{\left[0,1\right]}^{N}\to \left\{0,1\right\}.$$

This formulation is motivaged in Chandrasekaran et al. (2004) from a general perspective: “Real-world multidimensional signals, however, often consist of discontinuities separating smooth (but not constant) regions. This motivates a search for sparse representations for signals in any dimension consisting of regions of arbitrary smoothness that are separated by discontinuities in one lower dimension of arbitrary smoothness.” From this perspective, contour-surface factorization could be a general principle that is used by the cortex to deal with natural signals, and this bias could have been something discovered by evolution.

This innate bias of the visual system could also explain why humans are not very good at recognizing or remembering QR codes. While these two dimensional patterns are now ubiquitous because of the ease by which computer vision systems can reliable detect them, people find them hard to parse or remember. That would make sense, because a QR code is not a natural signal of the kind the human visual system has an innate bias toward. On the other hand, a convolutional neural network (CNN) can be trained to classify QR codes, or even noise patterns, and this could be indicative of the lack of human-like biases in a CNN.

#### 4.2.3. Computational and Algorithmic Perspective

Factorizations in concordance with the structure of the data allow for efficient learning, inference and generalization. Our hypothesis is that the blob-interblob structure in the visual cortex corresponds to a factorized representation of contours and surfaces (Figures 2C,D) similar to the Cartoon+Texture representation (Buades et al., 2010) proposed in image processing. Earlier computational realizations of this idea have include Markov Random Field models where the surface interpolation process and the boundary detection process are combined into an inter-active and concurrent system (Lee, 1995), or as an explicit neural network model (Grossberg, 2009).

### 4.3. Lateral Connections for Contour Continuity

#### 4.3.1. Biological Observation

**Figure 3:**
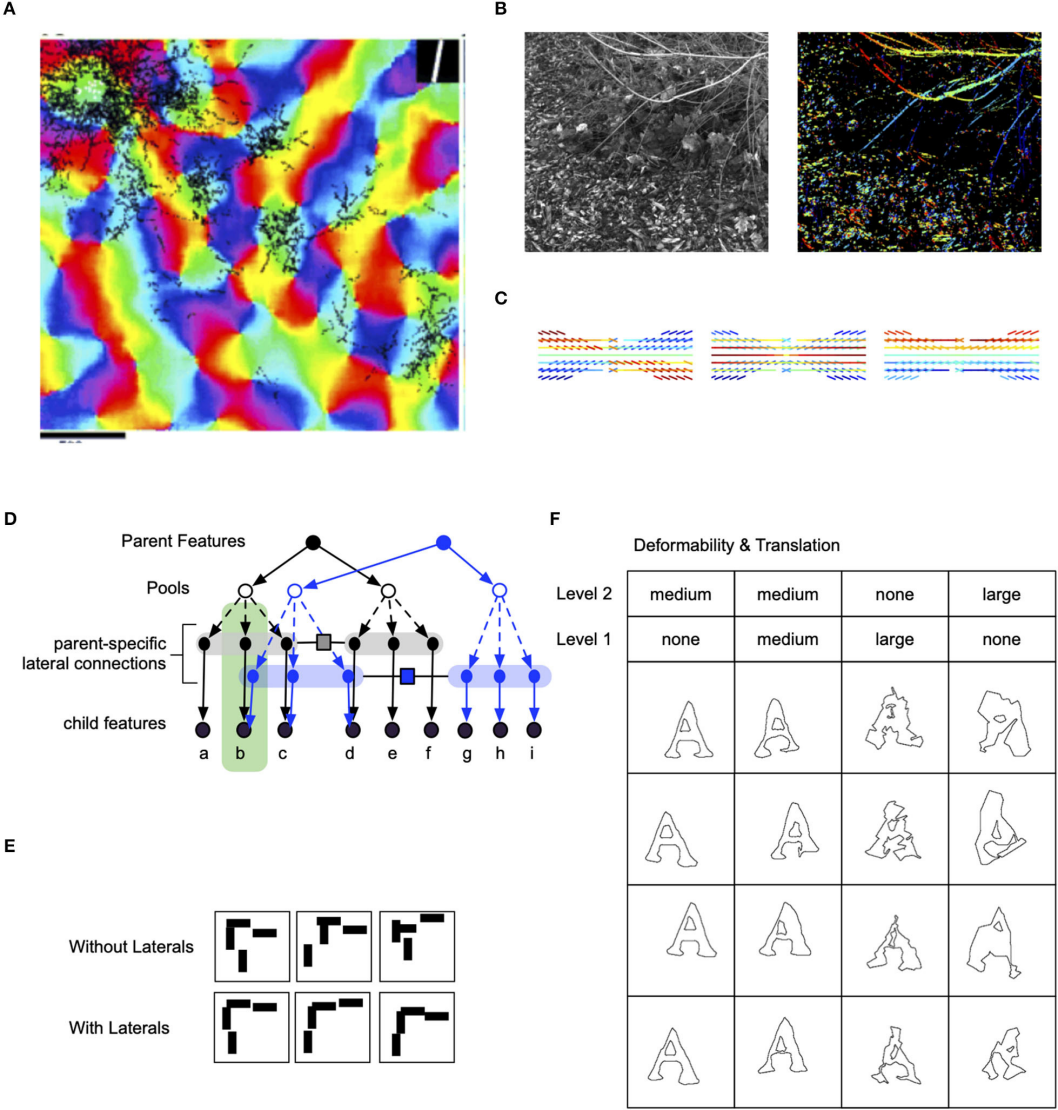
Lateral connections in the visual cortex and their computational significance. **(A)** Lateral connections project long distances and connect to columns that are of similar orientation (Bosking et al., 1997). **(B)** Analysing the co-occurrence statistics of contours in natural images show that they have higher-order structure than just pair-wise (image credit: Lawlor and Zucker, 2013). **(C)** Visualization of third-order structure in natural contours show that co-circularity and collinearity are represented (image credit: Lawlor and Zucker, 2013). **(D)** Lateral connections in RCN enforce contour continuity. **(E)** Samples with and without lateral connections. **(F)** The effect of flexibility of lateral connections at different levels in RCN.

Pyramidal neurons in the superficial layers of the visual cortex have extensive inter-columnar lateral projections. These connections link functionally similar regions: blobs are predominantly connected to blobs and interblobs are predominantly connected to interblobs (Yabuta and Callaway, 1998). Within the interblob subsystem, patches of intrinsic lateral connections tend to link columns sharing similar orientation preferences (Figure 3A) (Malach et al., 1993). In addition, neurophysiological experiments show that the neural responses to oriented bars contained within the classical receptive field are enhanced by coaxially placed flanking bars outside the classical receptive field (Hess et al., 2015).

Psychophysics observations show that contour integration is part of perception. When discrete line segments that follow Gestalt “good continuation” laws are embedded in a background of randomly oriented line segments, people easily group together the global contour (Hess et al., 2015). Computational researchers have suggested that long-range horizontal connections in the visual cortex are engaged in the geometrical computations required for contour integration (Hess et al., 2003; Ben-Shahar and Zucker, 2004) (Figure 3B).

#### 4.3.2. Property of World's Data

Contour continuity is an evident property of real-world objects, where sharp corners happen less frequently than smooth continuations. This is a particular case of the more general preference for smoothness that we find in real data. This is well-known by data science practitioners: when using a support vector machine or a Gaussian process, the most common non-linear kernel choice is the radial basis function. This choice (whose spectral representation is also a Gaussian) amounts to encoding a world in which low frequencies (smooth signals) tend to dominate.

#### 4.3.3. Computational and Algorithmic Perspective

Computationally, lateral connections and contour integration (Zhaoping, 2011) play an important role in hierarchical models with invariant representations by enforcing selectivity that is lost via the pooling operation. The Hubel-Wiesel model of stacking simple-cells for feature detection, and complex cells for translation pooling is the underlying mechanism for local transformation invariance in neocognitrons, HMAX-models, HTMs, and CNNs. However, uncoordinated pooling of features results in poor shape representations because the different components that constitute a higher-level feature can move independently (Geman, 2006). In a hierarchical model that hopes to achieve invariance through pooling needs to have lateral connections to enforce selectivity.

***Clonal neurons enable higher-order lateral interactions***. Clonally related excitatory neurons in the ontogenetic column are known to share similar physiological functions, such as visual orientation selectivity (Li et al., 2012; Ohtsuki et al., 2012). Recent studies by (Cadwell et al., 2020) suggest that, for pyramidal neurons in layer 2/3, shared input from layer 4 could be the source of similarity in orientation selectivity. Furthermore, these studies suggest that integration of vertical intra-clonal inputs with lateral inter-clonal input maybe a developmentally programmed connectivity motif.

RCN offers a potential explanation for the computational role of these clonal motifs. The contour integration association field need to capture long-range dependencies like curvature (Figure 3C), not just local collinearity provided by pairwise association field (Ben-Shahar and Zucker, 2004). The clonal neurons offer an efficient mechanism for capturing such long-term dependence. In RCN, coordinating receptive fields at different hierarchical levels is achieved by keeping separate copies (Figures 3D–F) of lateral connections in the context of different higher-level features; superposing these different lateral connections by marginalizing over the parent features would give rise to a pair-wise association field.

### 4.4. Hierarchy

#### 4.4.1. Biological Observation

It is well-established that the visual cortex is a hierarchy (Felleman and Van Essen, 1991). In the ventral stream, information gets passed successively through visual areas V1, V2, V4, and IT. The neurons in region V1 see only a small portion of the visual field. In general the receptive field sizes increase as you go up in the hierarchy. Physiological observations show that neurons in V1 respond to local oriented edges, or local luminance or color patches, whereas neurons in IT represent whole objects, with intermediate levels representing contours and object parts (Connor et al., 2007; Dicarlo et al., 2012).

#### 4.4.2. Property of the World

Natural and man-made dynamic systems tend to have a nested multi-scale organization, which might be a general property of all physical and biological systems. According to Simon (1973), building complex stable systems require the re-use of stable sub-systems that can be assembled together to build larger systems.

#### 4.4.3. Computational Perspective

According to the good regulator theorem (Conant and Ross Ashby, 1970), it would make sense that the visual system evolved to exploit the hierarchical structure of the visual world. By mirroring the hierarchical structure of the world, the visual cortex can have the advantage of gradually building invariant representations of objects by reusing invariant representations for object parts. Hierarchical organization is also suitable for efficient learning and inference algorithms.

### 4.5. Feedback Connections, Recurrent Processing, Inference, and Predictive Coding

#### 4.5.1. Biological Observations

Cortical connections are reciprocal. For every feedforward connection, there is a corresponding feedback connection that carries information about the global context. The origination, termination, and intra-columnar projections of feedback pathways follow layer-specific patterns that are repeated across multiple levels of the visual hierarchy (Thomson and Lamy, 2007). The feedback pathway modulate the responses of neurons in the lower-levels in myriad ways (Hochstein and Ahissar, 2002; Gilbert and Li, 2013). The feedback pathway, along with lateral connections, shape the tuning of the neurons beyond the classical receptive field by gradually incorporating global contextual effects through recurrent computation (Gilbert and Li, 2013). Feedback connections are implicated in texture-segmentation (Grossberg and Mingolla, 1985; Roelfsema et al., 1998), figure-ground separation (Hupé et al., 1998; Lamme et al., 1999), border-ownership computation (Von der Heydt, 2015), and object-based or feature-based attention (Tsotsos, 2008).

#### 4.5.2. Property of World's Data

Natural signals have a high amount of variation. The same underlying cause, the presence of an object, can manifest in many different ways in sensory data based on the location, viewpoint, lighting, shadows, and other influences on the scene. Any local observation about the world is likely to be ambiguous because of all the factors of variation affecting it, and hence local sensory information needs to be integrated and reinterpreted in the context of a coherent whole. Feedback connections are required for this.

#### 4.5.3. Computational and Algorithmic Perspective

Although there is rich data about the anatomy and physiology of feedback connections, their functional roles haven't been fully integrated into a model in the context of real-world problems. In RCN, feedback connections have three main roles:

***Vision as a generative model***. Feedback connections are key to treating perception as a generative model. Rather than treating vision as a feed-forward cascade of filters, as in a deep CNN, the generative model approach assumes that the brain is building a model of the causal processes underlying vision. In this perspective, perception is the process of inverting this process through inference. The generative model can be encoded efficiently in a probabilistic graphical model that mirrors the hierarchical organization of the world. Inference can be achieved efficiently through local message-passing algorithms like belief propagation (Pearl, 1988). In this setting feedback messages are just the top-down messages in a probabilistic graphical model.

**Figure 4:**
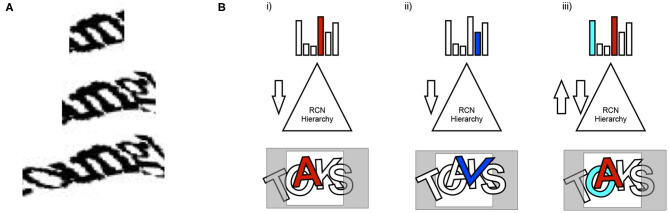
The need for explaining away in parsing visual scenes. **(A)** Local evidence suggests that the character is “m.” Incorporating global context shows that “un” is a better explanation. Even if “m” is strong in a feed-forward pass of inference, its evidence needs to be explained-away. **(B)** Feedforward-feedback iterations are used to explain away evidence to arrive at the globally best solution. Alternative partial explanations are hallucinated in the process of analyzing the scene.

***Explaining away and resolving ambiguity***. A general principle of vision is that local information is ambiguous and that it needs to be integrated and explained in terms of the global context. This is true even for CAPTCHAs. The ambiguity of local evidence is not just at the local edge or local contour level—even character-level percepts that make sense locally could be in conflict with the best global explanation. To evaluate the evidence in a scene to arrive at the approximate best global explanation (approximate MAP solution), local evidence has to be properly 'explained away' in the global context (Figure 4).

***Binding based on top-down attention***. An object is not always presented as contiguous in space. Noise and occlusion can separate out the parts of an object, but visual perception can bind those parts together into a coherent whole. Feedback and lateral propagation is required to support this kind of binding. When multiple objects with different attributes (for example, shape and color) are present in a scene, the attributes need to be bound to the right object. Top-down attention enables this kind of binding.

Top-down object-based attention is not based on spatial separation of objects. It works even when objects are completely overlapping in space. Being able to bind the contours and attributes of overlapping objects imposes some architectural constraints on the model. In RCN, these requirements translate to positive only weights, and lateral connections.

### 4.6. Bringing It All Together: Structured Probabilistic Model With Belief Propagation for Inference

We discussed some example representational principles and model constraints that can be learned from the brain by utilizing the triangulation strategy we outlined earlier. These principles are interconnected with each other, and the mutual constraints that they offer can help in figuring out the whole puzzle just like the different pieces of a jigsaw puzzle. Building a unified model that brings all these principles together, testing them on real world data, and iterating to improve the model and expand its capabilities is one way in which we can work toward building general intelligence. In this section we make a few remarks about building the joint model.

#### 4.6.1. Unified Vision Model, Multi-Task Performance, and Out-of-Distribution Generalization

**Figure 5:**
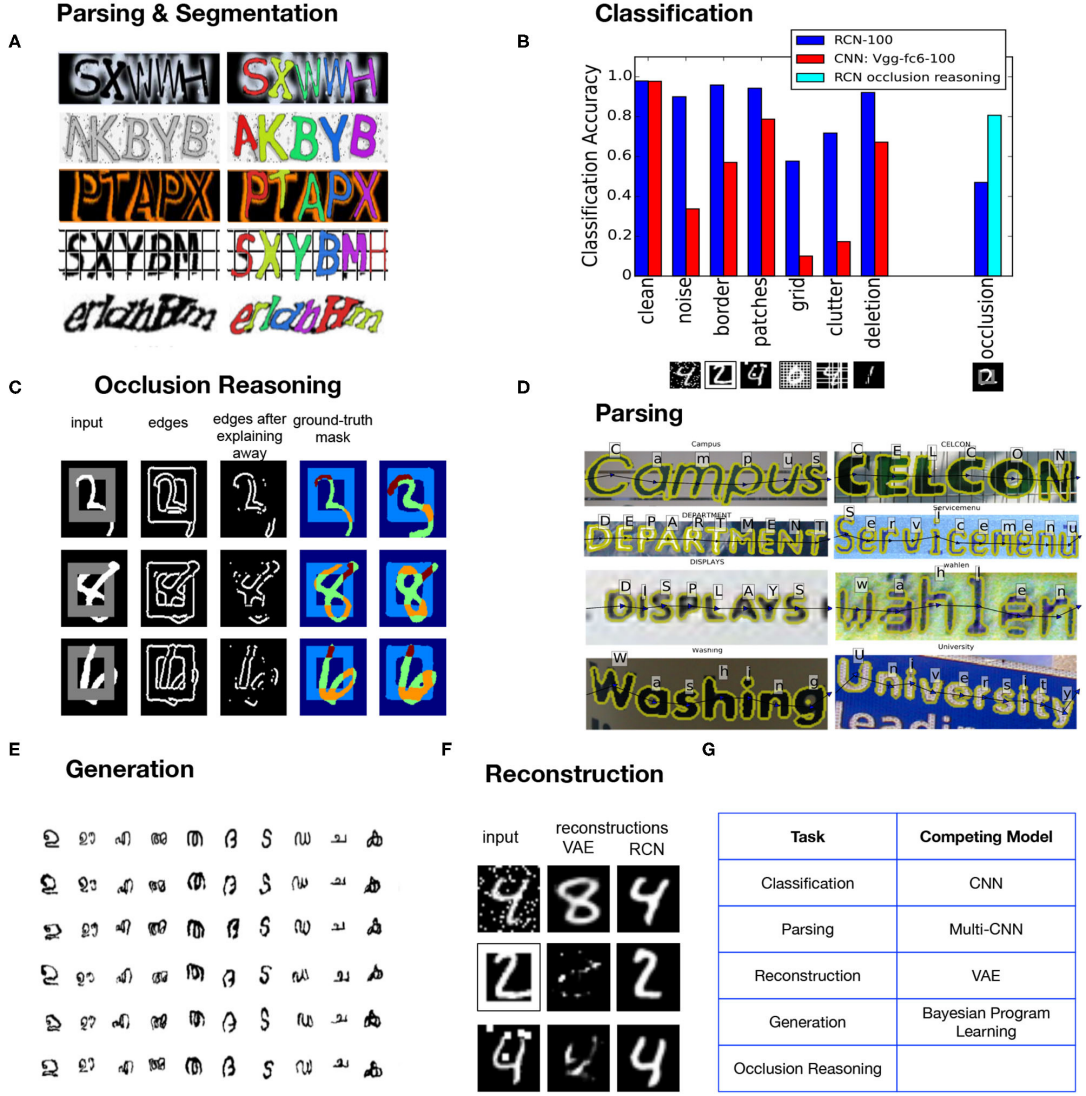
**(A)** Object recognition and object segmentation are two entangled problems for which a joint solution is necessary. **(B)** Test data with types of noise that were never seen during training degrade the performance of a CNN significantly more than RCN. **(C)** Occlusion reasoning increases the performance of RCN when objects overlap and allows hidden edges of an object to be recovered. **(D)** An example of parsing by RCN on a real world image. **(E)** RCN can render novel variations of characters after one-shot training. **(F)** A variational autoencoder and RCN try to reconstruct a digit in the presence of some type of noise that it was not trained for. **(G)** RCN is a single vision model that can perform all the required functions, as opposed to a collection of different models solving each of them.

Solving text-based captchas was a real-world challenge problem selected for evaluating RCN because captchas exemplify (Mansinghka et al., 2013) the strong generalization we seek in our models—people can solve new captcha styles without style-specific training. In addition, we tested RCN for multiple tasks, such as classification, segmentation, generation, reconstruction, in-painting, and occlusion-reasoning—all using the same model, and without task-specific training (Figure 5). We then compared its performance against models that are optimized for the specific tasks. Moreover, in each of these tasks we tested for data-efficiency and for generalization out of the training set distribution. Building a unified model might have the short term disadvantage of not being the best compared to models that are directly optimizing the task-relevant cost function, but in the longer term these models are likely to win out due to their data efficiency and strong generalization.

#### 4.6.2. Message-Passing Inspired by Cortical Dynamics

RCN was instantiated as a probabilistic graphical model (PGM) (Pearl, 1988). PGMs provide a rigorous framework for specifying prior knowledge, and uncertainty and inference are first class citizens of the framework. Moreover, graphical models allow the encoding of causality (Pearl, 2000). However, inference in complex PGMs like RCNs can be very challenging. There are no efficient algorithms that are theoretically guaranteed to give even approximately correct answers when the PGMs have multiple loops as is the case for RCNs.

The speed of cortical dynamics in visual perception points to message-passing algorithms (Pearl, 1988; Lee and Mumford, 2003) as a plausible mechanism for inference. In addition to the structural constraints learned from neuroscience, the representational choices in RCN were determined under the constraint that accurate inference should be possible using message-passing. Scheduling of the messages have a significant influence on the convergence and accuracy of loopy belief propagation messages, and we found that cortical dynamics (Lamme et al., 1999; Lee and Nguyen, 2001) can be used as a guide to design an advantageous propagation schedule. Such constraints could be useful for future modeling as well.

#### 4.6.3. Connections to Predictive Coding and the Free Energy Principle

RCN is compatible with the overarching frameworks of Free Energy Principle (Friston, 2010), and the observations regarding predictive coding in the brain. Some variants of message-passing algorithm are known to minimize approximations of the Bethe free energy (Yedidia et al., 2001). The top-down messages in RCN can be thought of as predictions in a predictive coding model. Many predictive coding models assume that the predictions need to be subtracted from the bottom-up input, but those assumptions are valid only in settings similar to a Kalman filter model (Friston, 2010). RCN uses a more generalized version of predictive coding where top-down messages are combined with bottom-up evidence in the appropriate way depending on how multiple causes interact to generate the data.

## 5. Discussion: Open Questions About Building AGI

### 5.1. Is AGI Even Possible? Shouldn't It Be Called AHI?

We consider Artificial General Intelligence (AGI) to be the artificial instantiation of human-like general intelligence. When we understand the general principles behind the operation of the human brain, we will be able to build machines that learn and generalize like it, and that will be AGI. We know we can build it because there is an existence proof.

Why don't we call it Artificial Human Intelligence (AHI) then? Since we are constructing this artificially, it will not have some of the biology-induced arbitrary constraints of the human brain. Perhaps our implementation can have unlimited working memory. Perhaps our implementation can have a fast internet interface directly hooked in. By instantiating the principles of intelligence in a different substrate, we already make it more general than AHI, which would be an exact replica of human-like intelligence.

We know that human-intelligence exists, but that doesn't mean AHI can be built. Are we really interested in putting in all the constraints and the same embodiment in our intelligent machines so that we create AHI, and not AGI? We wager that it would be simpler to create AGI.

Our brains are general, but that generality has limits. The success of biological evolution in creating our brains is not an existence proof for the same process resulting in an arbitrarily powerful intelligence [call it Artificial Universal Intelligence (AUI)]. Like perpetual machines, AUI is easy to imagine, but infeasible to build because it depends on physically impossible constructs like infinite computing power or infinite amounts of data. Delineating the limits of generality of human intelligence, and helping to understand which of those limits are fundamental algorithmic limits as opposed to arbitrary hardware constraints that biology had to adhere to is another way in which cognitive science and neuroscience can help AI research.

### 5.2. Don't We Need a Precise Mathematical Definition of AGI to Build One?

Researchers sometimes get hung up on the definition of intelligence, and some argue that without a well-accepted definition, progress cannot be made and the problem cannot be worked on. We disagree with this characterization.

What was the definition of the first iPhone? Complex products like iPhone do not get built from a one-sentence, or multi-sentence definition. Instead, they are built from functional requirements, that are also updated iteratively. Not all functional requirements are nailed down up front. Prototypes are built and iterated on, and the functional requirements change based on the built prototypes.

AGI does have functional specifications. We can reference the learning, acting, and reasoning abilities of children to understand what the functional requirements of an AGI are, and productive research programs can be built based on those. Just like the built iPhone defines what the product is, the final output of this research process will be the definition of AGI.

### 5.3. Shouldn't We Build a Worm-Level or Wasp-Level Intelligence Before Building AGI?

Spiders, worms, insects—they all exhibit very sophisticated behavior using biological neural networks that are vastly simpler than our brains. How do they do this? We don't even understand these simple neural circuits, so how could we understand our more complex brain? Shouldn't we try to reverse-engineer the neural circuit of a simpler animal, a spider, before we take on the complexity of the mammalian brain? While this is a legitimate question, the evolutionary history of intelligence described in section 2, suggests that a path to general intelligence do not necessarily need to go through these steps. We know that evolution can produce sophisticated, but idiosyncratic, circuits that function well in ecological niches. Reverse engineering the circuit for a specific organism can be a very hard task in itself, and achieving that might not give us much insight into the function of another organism with a different circuit. In contrast, the general uniformity of neocortex, and the preserved brain structures across different species, can provide an easier path for identifying general principles of general intelligence.

### 5.4. Are Artificial Neural Networks the Best Model Class for Building Brain-Like Intelligence

While artificial neural networks are excellent at function approximation, building general intelligence requires model building, inference to best explanation, and causal inference. These are not natively supported in artificial neural networks. On the other hand causal graphical models, and probabilistic programming offer sophisticated tools that allow for model building and inference. However, learning complex graphical models remains a significant challenge. While ANN function approximators can be learned with minimal set of assumptions, it is possible that learning graphical models will require much more inductive biases and structural assumptions than that are used by contemporary machine learning researchers. Our view is that the solution for AGI will need to combine tools from graphical models, causal learning and inference, program learning, and structure search, in addition to gradient-based optimization. The representational structure might to be graphical, with accelerated learning and inference obtained by combining it with neural networks (Lázaro-Gredilla et al., 2020).

### 5.5. Do Hybrid Models Imply Neural Networks for Perception and Symbols on Top of Neural Networks for Reasoning?

One of the shortcomings of neural networks is the difficulty in obtaining systematic generalizations that are explicit in factorizations in graphical models or in symbolic structures. Several researchers have suggested that the final solution for general intelligence will have components that are neural-net-like and components that are symbol manipulation-like. One take on this is that neural networks will solve the pixel-to-symbol problem, and that symbol-problems are then handled by a symbol manipulating model, a view popularized as system-1 and system-2 in *Thinking Fast and Slow* by Kahneman (2011). We take the position that such strict separation between perception and cognition is unlikely to be true. Problems in perception still need dynamic inference, which means that the reasoning components will need to go all the way down to sensory regions, so that perception and cognition can work together. In our opinion, hybrid models are more likely to be a combination of graphical models, graph-structured neural networks, causal inference, and probabilistic programs (Lázaro-Gredilla et al., 2019). Neural networks will help to accelerate inference and learning in many parts of these hybrid models. In that perspective, system-1 and system-2 are different modes of inference on the same underlying model rather than two separate systems.

## 6. Conclusion

The brain is often touted in articles about AI as a source of inspiration. However, the development process of new AI algorithms or techniques is usually the opposite: solving a task is used as a guide, and only then parallels with the brain are sought for. A prime example of this are neural networks. Despite their name, they were developed to solve the problem of curve fitting, and it was only after they were successful at this task that researchers started looking for ways in which the brain could be biologically implementing them (Lillicrap et al., 2020). Finding this biological support after the fact is indeed interesting, but mostly inconsequential to the practical success and impact of NNs.

In this work we claim that observing that AGI and the brain are connected is not enough to make progress in the former, and note several common pitfalls in the search for AGI, as well as avenues for success.

A common pitfall is thinking that state-of-the-art task solving can be equated to intelligence. Just because an approach is solving a problem very well, it does not mean that the approach is intelligent or taking us closer to intelligence. AGI will be characterized by its generality at accomplishing a wide range of tasks, and not by excelling at each and all of them. For each task that AGI solves, we can expect a non-AGI solution to outperform it.

A more fruitful approach might be to consider simultaneously the insights from neuroscience (e.g., the factorized representations of contours and surfaces), general real world properties (objects actually being efficiently describable as cartoon + texture), and the computational efficiency of the corresponding models (more efficient learning and generalization in models with this factorization). When triangulating between these three elements, new computational techniques can be devised. These might be more likely to unlock generally applicable principles that take us closer to AGI.

We have provided RCN as a concrete computational example of success in this triangulation process. While it is relatively easy to postulate abstract principles, we believe that it is more inspiring to see how the whole process plays out all the way to a concrete computational model that can tackle different vision problems in an integrated manner.

Finally, we discuss some open questions about building AGI and present arguments against some of them that are prevalent in today's mainstream approaches toward AGI. For instance, thinking that defining AGI mathematically is needed to be able to develop it is usually hindering the progress in AGI instead of fostering it. And the assumption that feed-forward NNs (and in particular deep versions of it) are the best substrate for AGI is devoting too many resources to a particular computational model that can be easily shown to not contain some of the key elements required for AGI.

## Author Contributions

DG conceived the paper and wrote the paper. ML-G wrote the sections of the paper. JG wrote the sections of the paper. All authors contributed to the article and approved the submitted version.

## Conflict of Interest

DG, ML-G, and JG are employed by the company Vicarious AI.

## Footnotes

^1^Something similar happens when evolutionary algorithms are used to discover electronic circuits. The evolved circuits use clever coupling between transistors that might be idiosyncratic to a particular wafer.

^2^If survival and dominance are what is needed, brute power and instincts can go very far without great thinking capabilities.
